# Molecular Diagnosis of Malaria by Photo-Induced Electron Transfer Fluorogenic Primers: PET-PCR

**DOI:** 10.1371/journal.pone.0056677

**Published:** 2013-02-20

**Authors:** Naomi W. Lucchi, Jothikumar Narayanan, Mara A. Karell, Maniphet Xayavong, Simon Kariuki, Alexandre J. DaSilva, Vincent Hill, Venkatachalam Udhayakumar

**Affiliations:** 1 Division of Parasitic Diseases and Malaria, Center for Global Health, Centers for Disease Control and Prevention, Atlanta, Georgia, United States of America; 2 Waterborne Disease Prevention Branch, National Center for Emerging and Zoonotic Infectious Diseases, Centers for Disease Control and Prevention, Atlanta, Georgia, United States of America; 3 Atlanta Research and Education Foundation/VA Medical Center, Decatur, Georgia, United States of America; 4 Malaria Branch, Kenya Medical Research Institute/Centers for Disease Control and Prevention, Kisumu, Kenya; Royal Tropical Institute, Netherlands

## Abstract

There is a critical need for developing new malaria diagnostic tools that are sensitive, cost effective and capable of performing large scale diagnosis. The real-time PCR methods are particularly robust for large scale screening and they can be used in malaria control and elimination programs. We have designed novel self-quenching photo-induced electron transfer (PET) fluorogenic primers for the detection of *P. falciparum* and the *Plasmodium* genus by real-time PCR. A total of 119 samples consisting of different malaria species and mixed infections were used to test the utility of the novel PET-PCR primers in the diagnosis of clinical samples. The sensitivity and specificity were calculated using a nested PCR as the gold standard and the novel primer sets demonstrated 100% sensitivity and specificity. The limits of detection for *P. falciparum* was shown to be 3.2 parasites/µl using both *Plasmodium* genus and *P. falciparum-*specific primers and 5.8 parasites/µl for *P. ovale*, 3.5 parasites/µl for *P. malariae* and 5 parasites/µl for *P. vivax* using the genus specific primer set. Moreover, the reaction can be duplexed to detect both *Plasmodium* spp. and *P. falciparum* in a single reaction. The PET-PCR assay does not require internal probes or intercalating dyes which makes it convenient to use and less expensive than other real-time PCR diagnostic formats. Further validation of this technique in the field will help to assess its utility for large scale screening in malaria control and elimination programs.

## Introduction

Four different *Plasmodium* species with different clinical implications infect humans in different combinations around the world, with recent studies demonstrating that *P. knowlesi* is also capable of causing human malaria due to zoonotic transmission [1]. Approximately 225 million cases of malaria and 781,000 deaths due to malaria occurred in 2009 [2]. Due to the apparent decline in the number of malaria cases and deaths in the last few years, there is a concerted global effort to control and eliminate malaria with the support of many public and private initiatives (reviewed in [3], [4]). Therefore, there is a clear need for diagnostic tools that are robust enough to accurately detect the species of infecting parasite(s), to identify the transmission foci of malaria reservoirs (often which may be submicroscopic and asymptomatic) and to monitor the success of malaria control and elimination programs.

The existing tools for malaria diagnosis include microscopy, parasite antigen/enzyme detection kits [commonly referred to as rapid diagnostic tests (RDTs)] and molecular tools (nucleic acid based tools). Microscopy remains the gold standard for the diagnosis of malaria in many malaria endemic countries. Although microscopy is relatively inexpensive and can be used to differentiate parasite species as well as provide quantitative data on the level of parasitemia, several limitations including poor sensitivity [5]–[9] begs for novel methods in the elimination era. RDTs have become an alternative tool for malaria diagnosis. While some RDTs are *Plasmodium*-specific, (pan) detecting the genus-specific aldolase and lactate dehydrogenase enzymes, the majority of the RDTs are specific for the *P. falciparum* histidine-rich protein –2 (Pf HRP-2). The recent discovery that some *P. falciparum* parasites in parts of South America and Africa have deleted the *hrp-2* gene [10], [11] has brought into question the use of the HRP-2 based RDT tests due to potential false negatives. Another drawback of RDTs is the fact that they cannot be used to determine parasite densities and such quantification is increasingly essential with respect to both, antimalarial drug resistance surveillance and malaria control programs. With limits of detection at ∼100 parasites/µl (reviewed in [12]), RDTs can only be useful for routine diagnosis and treatment. With the limitations of both microscopy and RDTs, there is a clear realization that more sensitive diagnostic tools are needed for the detection of subclinical but transmissible infections to guide and monitor malaria elimination programs [13].

Molecular tools are far more sensitive in detecting low level infections and accurately detecting species of malaria parasite (reviewed in [5], [12]). There are many versions of molecular tools for malaria diagnosis ranging from conventional PCR-based assays, real-time PCR assays and more recently, isothermal amplification assays (reviewed in [7], [12]). When considering the development of tools for large scale field application, it is desirable to consider cost, robustness, and ease of use. The real-time PCR platform has some advantages for large scale screening as it requires no major post-PCR steps to acquire the results and a large number of samples can be analyzed at one time.

Real-time PCR assays depend on the use of fluorophores or DNA intercalating fluorescent dyes. As in the cases of TaqMan probes, molecular beacons and scorpion, sequence-specific oligonucleotide probes are dual labeled with a fluorescent dye and quencher. Although these molecular methods are robust and simpler than conventional PCR methods for large scale screening, the reagents are expensive and require special handling, which then creates practical challenges for field use in malaria endemic countries. A less complicated real-time PCR technique is the use of non-specific DNA intercalating dyes such as SYBR Green, SYTO-9, and calcein, which emit fluorescence signals when bound to double stranded DNA. Another drawback for these non-specific intercalating dyes is that they do not allow for multiplexing. Other alternatives for the detection of real-time PCR assays have been described including the direct labeling of one of the primers (forward or reverse) with a single fluorophore in a manner that facilitates self-quenching without the need of a quencher. These self-quenching primers facilitate the use of real-time PCR without the need for internal dual-labeled sequence specific probes. An example of this is the Light Upon Extension (LUX) primer that is labeled with an internal single fluorophore near the 3′ end of the primer in a hairpin structure which allows for the quenching before amplification [14]. These self-quenching, single labeled primers are less expensive compared to the dual labeled probe. However, the design of LUX primers requires the specific software, LUX Designer.

**Figure 1:**
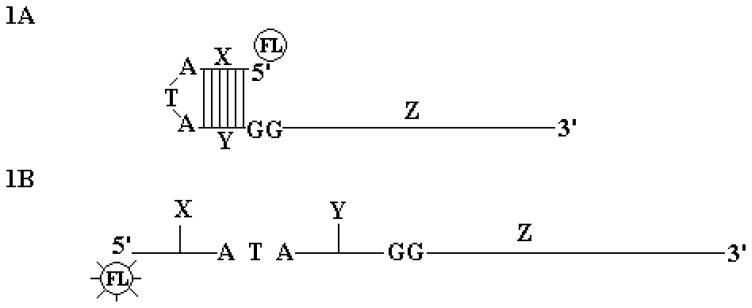
PET-PCR Primer design. The photo-induced electron transfer (PET) primer consists of a quasi-universal tail sequence at 5′ and sequence specific region at 3′. This 5′ generic tail sequence consists of a 6 base pair stem, 3 nucleotide loop, a single fluorophore attached at 5′ end and two deoxyguanosine located in the opposite overhang region. The first two dangling end base on the PET primers are deoxyguanosine located at first and second overhang position respectively provided the greatest quenching in the absence of amplification. Circled FL is fluorescence attached to 5′. Z is sequence specific primer. X, ATA and Y together form a loop like structure. X and Y are complementary structure. Figure.1B. Fluorescence of PET primer increases during amplification when an extension of the complementary strand takes place. The result of fluorescence increase is due to dequenching effect of deoxyguanosine located in overhang positions and due to the formation of double strand.

Here we describe a novel method for labeling real-time PCR primers based on the principle that was recently described in a patent filed by Jothikumar et al. (patent # PCT/US2008/084347). In this simplified technique, the 5′ end of one of the primers is modified by the addition of a 17-bases oligonucleotide tail that is labeled with a fluorophore (HEX or FAM) on its 5′ end, (Figure 1). In the absence of amplification, this tail forms a loop and remains in a closed conformation resulting in effective quenching of fluorescence due to close proximity of four G bases (i.e., the two overhang GG and two complementary GG residues in the hairpin formation) via photo-induced electron transfer (PET) mechanism; hence the name PET-PCR (not to be confused with the PET fluorescence dye from ABI). When participating in nucleic acid amplification, the stem loop structure of the PET primer opens up. The subsequent fluorescence increase is due to the de-quenching effect of the two guanosine residues located in the overhang positions and also due to the formation of the complementary DNA strand.

To evaluate this concept, we designed PET-PCR primers for the specific amplification of the *Plasmodium* genus and *P. falciparum* species. We demonstrate the utility of this assay using 119 clinical samples of different malaria species infections, including mixed infections, and using well quantified Plasmodium species to estimate the limits of detection of this assay.

## Materials and Methods

### Ethics Statement

Some samples used in this study were obtained from a study conducted in Kenya. This study was approved by both the Kenya Medical Research Institute (KEMRI) and the CDC Institutional Review Board and informed written consent forms were obtained from each subject.

### PET-PCR Primers

The design of the PET-PCR primers follows the same common rules for PCR primer design. In this study the genus specific primers were designed to amplify the 18S ribosomal RNA gene of *Plasmodium.* The primers were designed based on GenBank accession # GU815531; the *P. malariae* 18S small subunit ribosomal RNA gene. A BLAST search was performed to ascertain that the region selected for primer design was conserved in the rest of the Plasmodia species. The target for the *P. falciparum* specific primer set, Pfr364, was described previously [15]. In both cases, the 5′ end of the reverse primers was modified with the PET tag and labeled with FAM (for the genus) and HEX (for *P*. *falciparum)*. These oligonucleotide primers for *Plasmodium* spp. and *P. falciparum* are shown in Table 1.

**Table 1:** Oligonucleotide primer sequences used in the real-time PET-PCR assay.

	Sequence (5′-3′)	Position
**Plasmodium genus-specific 18S ssrRNA** *		
**Forward**	GGCCTAACATGGCTATGACG	305–324
**Reverse**	FAM-aggcgcatagcgcctgg CTGCCTTCCTTAGATGTGGTAGCT	395-372
***Plasmodium falciparum***#		
**Forward**	ACCCCTCGCCTGGTGTTTTT	
**Reverse**	HEX-aggcgcatagcgcctgg TCGGGCCCCAAAAATAGGAA	

The target independent tail (underlined) is attached to the 5′end of the target specific sequences and do not show any homology to *Plasmodium* spp. sequences.

* Position based on GenBank accession # GU815531 for 18S small subunit ribosomal RNA gene.

# Designed based on a previously described *P. falciparum* specific target, Pfr364 [15].

### *Plasmodium* Parasites and Clinical Samples

Different *Plasmodium* species available in our laboratories were used in this study: *P. falciparum* (3D7, FCR3, W2, D6, Dd2, V1-S, Nigeria, Santa Lucia), *P. vivax* (South Vietnam IV), *P. malariae* (Uganda I), *P. knowlesi* (Nuri) and *P. ovale* (Nigeria I). DNA from 50 clinical samples available from the CDC molecular diagnostic parasitology reference laboratory (Dr. A. DaSilva) (10 non-malaria samples, 13 *P. falciparum*, 9 *P. vivax*, 1 *P. malariae*, 11 *P. ovale, 2 P. falciparum/P. malariae, 1 P. vivax/P. ovale, 2 P. falciparum/P. ovale* mixed infections and 1 *P. knowlesi*; parasitemia data not available) were used. In addition, a set of 69 *P. falciparum* positive samples from a study in Kenya were tested anonymously (microscopic parasitemia range of 1230–231,040 parasites/µl).

### DNA Extraction

DNA was isolated from all the samples using a QIAamp DNA Mini Kit (QIAGEN, Valencia, CA-(Qiagen method)). The DNA was aliquoted and stored at −20°C.

### PET-PCR Method

Amplification of *Plasmodium* spp. or *P. falciparum* was performed in a 20 µl reaction containing 2X TaqMan Environmental Master Mix 2.0 (Applied BioSystems), 125 nM each forward and reverse primer, and 2 µl of DNA template. The reactions were performed under the following cycling parameters: initial hot-start at 95°C for 10 minutes, followed by 45 cycles of denaturation at 95°C for 10 seconds, annealing at 60°C. The correct fluorescence channel was selected for each fluorescently labeled primer set and the cycle threshold (CT) values recorded at the end of annealing step. Any sample with a CT value of 40.5 or below was considered positive. Both singleplex and multiplex assays were performed for all the samples.

### Nested PCR and Real-time PCR Methods for Comparison

Due to its superior sensitivity over microscopy and other molecular tests, a nested PCR was chosen as a reference test to calculate the sensitivity and specificity of the PET-PCR. The nested PCR was performed with primers and cycling conditions as described by Singh et al. [16] with some modifications. Reactions were performed in a 20 µl total volume containing 1X buffer, 2.5 mM MgCl_2_, 200 µM dNTPs, 200 nM primers, and 1.25 units of Taq Polymerase (New England Biolabs, Ipswich, MA). The PCR amplified material was analyzed using gel electrophoresis (2% gel) to visualize the bands of appropriate size. Since the PET-PCR assay is a real-time PCR assay, a well-known real-time PCR diagnostic method developed by Rougemont et al. [17] (here after referred as Rougemont real-time PCR) was chosen as an additional method for making a quantitative comparison. The Rougemont real-time PCR is a duplex PCR, capable of detecting the four human infecting *Plasmodium* species in a set of two simultaneous separate duplex reactions (*P. falciparum* duplexed with *P. vivax* and *P. malariae* duplexed with *P. ovale*). For the comparison in our study, only one duplex reaction was performed (*P. falciparum* with *P. vivax)*. This assay was performed as described by the authors. Briefly, a 25 µl reaction containing 12.5 µl of TaqMan Universal Master Mix (Applied Biosystems), 200 nM each of the *Plasmodium* specific forward and reverse primers and 80 nM of the species-specific probes was prepared. The assay was executed using the following cycling conditions: an initial step at 50°C for 2 min, 95°C for 10 min, and 45 cycles of 95°C for 15 s and 60°C for 1 min. A cut-off CT value of 40 was used to indicate a positive result.

### Test for Specificity, Sensitivity

The specificity of the primers was first tested by their ability to correctly amplify the gene of interest. *P. falciparum, P. vivax, P. ovale, P. knowlesi* and *P. malariae* DNA were used in the first round of testing. In addition, geographically-diverse strains for *P. falciparum* (3D7, FCR3, W2, D6, Dd2, and V1-S) and *P.vivax* were used to test for specificity of the primers.

### Analytical Sensitivity

The analytical sensitivity (limits of detection) of both the genus- and falciparum-specific assays was determined using two well-quantified *P. falciparum* strains (Nigeria and Santa Lucia). The recommended WHO protocol for standards preparation used for quality control of RDT tests was used to prepare the *P. falciparum* standards (http://www.wpro.who.int/sites/rdt/using_rdts/qa/lot_testing.htm). The *P. falciparum* parasites were at the ring or early trophozoite stage of development when the sample was used (to exclude multinucleated parasites contributing to quantitative bias). The percent parasitemia was determined by three expert microscopists by counting the number of parasites in approximately 2000 erythrocytes examined. The total number of erythrocytes/µl was measured directly using a coulter counter and the number of parasites/µl was calculated from the total number of RBCs/µl and the percent parasitemia obtained. This standard sample was then diluted from the initial parasitemia to 2000 parasites/µl using uninfected whole blood and was then serial diluted fivefold to 0.64 parasite/µl using a 250 µl volume. DNA was extracted from each dilution point using 200 µl of sample. The analytical sensitivity of the PET-PCR assay was compared to that of the nested PCR [16] and a previously reported real-time PCR assay [17]. In addition, the limit of detection of the genus primer set to detect *P. ovale, P. malariae* and *P. vivax* was tested. These samples were kindly given to us by Dr. John Barnwell, Malaria Branch, Centers for Disease Control and Prevention (CDC).

### Clinical Sensitivity and Specificity

The clinical sensitivity and specificity of the PET-PCR assay was calculated using 119 clinical samples: 69 microscopically positive *P. falciparum* samples from Kenya and 50 clinical samples available from the CDC molecular diagnostic parasitology reference laboratory which were previously tested for malaria using the Rougemont real-time PCR assay. Microscopy data was unavailable for these 50 clinical samples. A nested PCR assay [16] was used as the reference test. The percentage specificity and sensitivity were calculated as follows: Sensitivity = true positives/(true positives+false negatives) × 100. Specificity = true negatives/(true negatives+false positives) × 100. In addition, 95% Confidence Intervals (95% CI) for both sensitivity and specificity were calculated.

## Results

### Detection of Different *Plasmodium* Species Using the Genus-specific and *P. falciparum*-Specific PET- primers

In the initial round of testing we were able to amplify the five human-infecting *Plasmodium* species (*P. falciparum, P. vivax, P. malariae*, *P. knowlesi* and *P. ovale*) using the genus-specific primer set. The *P. falciparum* specific primer set amplified *P. falciparum* only. Both primer sets detected all the additional *P. falciparum* strains tested (3D7, FCR3, W2, D6, Dd2, and V1-S). In addition, the genus-specific primers detected 11 strains of *P. vivax* and 5 simian Plasmodium species, *P. cynomolgi, P. inui, P. simiovale, P. hylobati* and *P. coatneyi*.

### Limits of Detection of PET-PCR Assay

The limits of detection of the genus and *P. falciparum*-specific primer sets was tested using serial dilution of calibrated DNA stock prepared from two *P. falciparum* strains (Nigeria and Santa Lucia). The parasitemia ranged from 2000, 400, 80, 16, 3.2 and 0.64 parasites/µl which was diluted using whole blood and DNA extracted for analysis. This was compared in parallel with the Rougemont real-time PCR and the nested PCR. Using a CT value of 40 as a cut off, both the PET-PCR and Rougemont real-time PCR assays detected as low as 3.2 parasites/µl. This was comparable to the nested PCR which consistently detected 3.2 parasites/µl and occasionally as few as 0.64 parasites/µl. The limits of detection of the genus-specific primer set for the other Plasmodium species was ascertained with DNA obtained from *P. vivax, P. ovale and P. malariae* infected blood and determined to be 5.0 parasites/µl, 5.8 parasites/µl, and 3.5 parasites/µl, respectively.

### Quantitative Comparison of PET-PCR Assay and Rougemont Real-time PCR Assay

**Figure 2:**
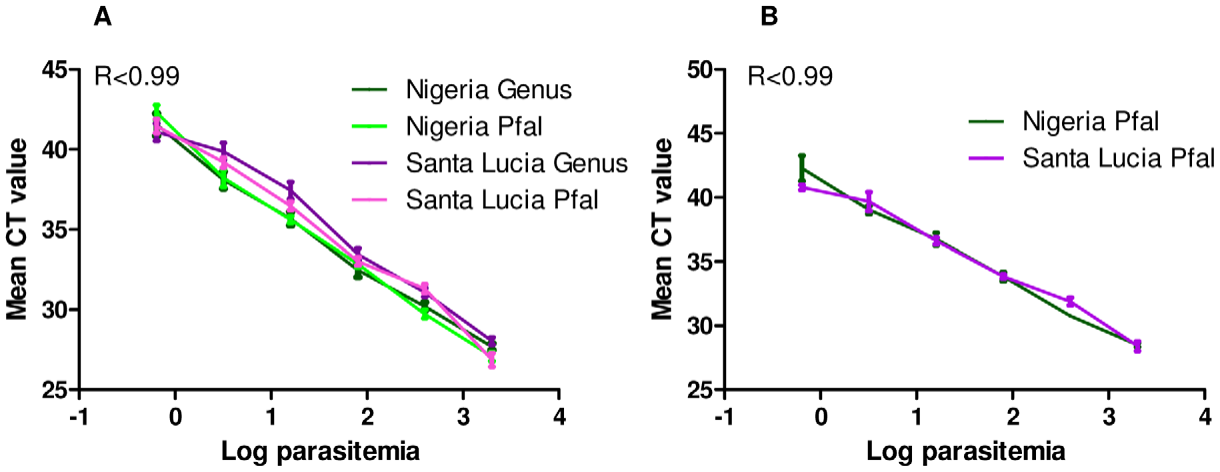
Quantitative comparison of PET-PCR assay and Rougemont real-time PCR assay. The PET-PCR and Rougemont real-time PCR assays were run using two well-quantified *P. falciparum* strains (Nigeria and Santa Lucia) used at six differing parasitemia levels (2000, 400, 80, 16, 3.2 and 0.64 parasites/µl). To determine the correlation between CT values and parasitemia, the mean CT values obtained in the PET-PCR assay (A) and in the Rougemont real time PCR assay (B) were plotted against the log transformed parasitemia (Log parasitemia). The CT values demonstrated reproducible linearity over the parasitemia range tested as both methods show significant correlation with R^2^ values >0.990. No statistical difference in mean CT values was observed for the two different strains or for the two different methods (P>0.05).

The quantitative nature of the PET-PCR method was compared in parallel with the Rougemont real-time PCR using the *P. falciparum* serial dilutions utilized in testing the limits of detection above. The obtained CT values were plotted against the log transformed parasitemia to determine any correlation between the two (Figure 2). Similar to the Rougemont real time PCR assay, the CT values obtained in the PET-PCR assay demonstrated reproducible linearity over the range of parasitemia tested in four replicate experiments. Both methods showed significant correlation with R^2^ values >0.990 and a significant correlation coefficient (p<0.0007) for the mean CT values and parasitemia implying that this method can be used to quantify parasitemia. No statistical difference in mean CT values was observed for the two different strains or for the two different methods (P>0.05).

### Multiplex Assay

To test the possibility of multiplexing the assay, the two primer sets were used in a duplex assay. A well-quantified *P. falciparum* strain (Nigeria strain) was used at six differing parasitemia levels (2000, 400, 80, 16, 3.2 and 0.64 parasites/µl). The reactions were performed both in the singleplex and multiplex formats. Both the singleplex and multiplex formats gave similar CT values for the different parasitemia levels tested, Table 2. In addition, the assay was performed using mock-mixed infections of *P. falciparum/P. vivax, P, falciparum/P. ovale and P. falciparum/P. malariae* with varying parasite densities of the *P. falciparum.* Both primer sets were able to detect all the varying concentrations in the mock mixed infections, Table 3.

**Table 2:** Reproducibility of the singleplex and multiplex PET-PCR assays for *P. falciparum* detection.

	Mean CT value ± SD					
Target	2000 (parasites/µl)	400(parasites/µl)	80(parasites/µl)	16(parasites/µl)	3.2(parasites/µl)	0.64(parasites/µl)
Genus-S	29.19±0.40	31.70±0.67	33.92±0.69	36.69±0.57	39.56±1.20	42.92±1.02
Pf- S	28.22±0.58	31.11±0.36	33.33±0.64	35.71±0.39	37.79±0.49	42.77±0.77
Genus-M	28.15±0.46	30.50±0.88	33.04±0.66	35.69±0.31	38.54±1.23	41.89±1.23
Pf-M	27.64±1.41	30.32±0.63	33.06±0.46	35.55±0.47	37.97±0.45	42.40±0.81

A well-quantified *P. falciparum* strain (Nigeria strain) was used at six differing parasitemia levels (2000, 400, 80, 16, 3.2 and 0.64 parasites/µl). The reactions using the genus-specific primers (Genus) and *P. falciparum*- specific primers (Pf) were performed both in the singleplex (Genus-S and Pf-S) and multiplex (Genus-M and Pf-M) formats. The mean CT values ± standard deviation (SD) obtained from three experiments each performed in duplicate are shown.

**Table 3:** Detection of mock mixed infections using the multiplex assay.

*P. falciparum/P. malariae* (Parasites/µl)	*P. falciparum (CT ±SD)*	Genus (CT ±SD)
**2000/7**	29.62±0.76	29.07±0.27
**400/14**	31.80±0.30	31.29±0.31
**80/28**	34.63±0.33	33.56±0.23
**16/280**	36.21±0.37	31.01±0.28
**3.2/2800**	38.61±0.53	27.80±0.21
**NTC**	No CT	No CT
***P. falciparum/P. vivax*** ** (Parasites/µl)**	***P. falciparum (CT ±SD)***	**Genus (CT ±SD)**
**2000/5**	29.98±0.18	29.46±0.44
**400/10**	31.88±0.43	31.63±0.15
**80/100**	34.23±0.33	33.35±0.85
**16/1000**	36.77±0.14	30.99±0.16
**3.2/2000**	39.70±0.84	27.57±0.53
**NTC**	No CT	No CT
***P. falciparum/P. ovale*** ** (Parasites/µl)**	***P. falciparum (CT ±SD)***	**Genus (CT ±SD)**
**2000/5.8**	29.56±0.29	29.31±0.31
**400/11.6**	31.01±0.13	30.41±0.47
**80/23.1**	34.08±0.42	32.92±0.41
**16/231**	35.51±0.39	30.92±0.27
**3.2/2300**	38.79±0.39	27.28±0.54
**NTC**	No CT	No CT

Mock mixed infections were prepared using a well-quantified *P. falciparum* strain (Nigeria strain) at five differing parasitemia levels (2000, 400, 80, 16 and 3.2 parasites/µl) combined with varying parasite densities of *P. malariae, P. vivax* and *P. ovale* (as shown in the table). The mean CT values for the *P. falciparum* and genus primers sets obtained from two experiments each performed in duplicate are shown. The multiplex assay was able to detect all the varying combinations of the mock-mixed infections. NTC =  no template control; SD = standard deviation.

### Clinical Sensitivity and Specificity of the *Plasmodium* Genus and *P. falciparum* Specific PET-primers

One hundred and nineteen clinical samples consisting of different malaria species and mixed infections were used to test the utility of the novel PET-PCR primers in diagnosis of clinical samples. As shown in Table 4 all the *P. falciparum* samples tested were shown to be positive by the *P. falciparum*–specific and the *Plasmodium* -specific primer sets. The genus-specific primers detected all other non-falciparum samples tested. No signal was detected with the 10 non-malaria human DNA. The sensitivity and specificity of these singleplex assays was calculated using the nested PCR as a reference test. Both primer sets showed 100% sensitivity and specificity. In the multiplex assays, the genus*–*specific primers were able to accurately detect all the *Plasmodium* spp. samples; however, the *P. falciparum*-specific primer set failed to detect two *P. falciparum* samples (Table 4). Microscopy data was only available for 69 *P. falciparum* samples. These samples (parasitemia range of 1230–231,040 parasites/µl) were all shown to be positive by both primers sets.

**Table 4:** Comparison of PET–PCR to nested PCR using clinical samples.

Number of samples detected by each method (n)				
Nested PCR	Singleplex-PET		Multiplex-PET	
	*P. falciparum*	Genus	*P. falciparum*	*Genus*
*P. falciparum* (82)	80/80#	81/81#	81/82*	82/82
*P. vivax* (9)	0/9	9/9	0/8#	8/8#
*P. ovale* (11)	0/9#	9/9#	0/11	11/11
*P. malariae* (1)	0/1	1/1	0/1	1/1
*P. knowlesi* (1)	0/1	1/1	0/1	1/1
*Pf/Po* (2)	2/2	2/2	1/2*	2/2
*Pf/Pm* (2)	2/2	2/2	2/2	2/2
*Pv/Po* (1)	0/1	1/1	ND	ND
Non-malaria (10)	0/8#	0/9#	0/10	0/10

A total of 119 clinical samples consisting of different malaria species and mixed infections were used to test the utility of the PET-PCR primers in diagnosis of clinical samples.

# Not all the samples tested by nested PCR were tested in all the PET-PCR assays due to insufficient sample volumes.

* The *P. falciparum* primer (used in the multiplex assay) failed to detect two *P. falciparum* positive samples.

ND =  not done.

## Discussion

The use of self-quenching real-time PCR primers using a 17-base loop tail was recently described in a patent filed by Jothikumar et al. (patent # PCT/US2008/084347). In this proof-of-concept study, we designed PET-PCR primers for the detection of *Plasmodium* spp. and *P. falciparum* and successfully demonstrated that these primers can be duplexed to detect both *Plasmodium* genus and *P. falciparum* species in a single tube. The sensitivities of this assay are better than those reported for microscopy and RDTs (reviewed in [18]), and comparable to that of other real-time PCR assays (range from 0.7–10 parasites/µl) [17], [19], [20]. As reviewed by Okell [5], the existence of submicroscopic infections is well documented and are known to contribute to the infective reservoirs of malaria gametocytes [21]–[23]. Therefore, sensitive assays such as the PET-PCR described here, that are capable of detecting submicroscopic infections are valuable for any malaria control and elimination programs, as these will help monitor transmission foci and dynamics as well as control efforts. Moreover, this assay provides the option of analyzing a large number of samples in a short time.

We demonstrate that PET-PCR is comparable to Rougemont real-time PCR (a Taqman based assay which is widely used for malaria diagnosis) both in terms of high sensitivity and the ability to quantify parasitemia. To date, no self-quenching real-time PCR assay has been described for malaria diagnosis. The self-quenching in PET-PCR facilitates the use of real-time PCR without the need for probe designs involving internal quenching thereby reducing the cost of the assay by obviating the need for expensive probes from commercial vendors. In addition, high-performance liquid chromatography (HPLC) purification is not required for the single labeled primer, resulting in a high yield of product further reducing the costs as compared to the commonly used dual-labeled probes. Furthermore, the PET-PCR primers can be either stored in the refrigerator without loss of activity for up to a month or be lyophilized and reconstituted when the end user is ready to use them. These practical advantages together with the lower costs and the easy design of PET primers (no specific software is required) offers promising features that can enable the adoption of this method as an alternate real-time PCR for malaria detection for large scale screening of field samples in large malaria surveillance and control programs.

The genus-specific assay described here offers an efficient, high-throughput malaria detection tool, ideal for the screening of malaria, irrespective of the species. This is useful, for example, in surveillance/screening epidemiological studies. It can be used to both monitor and evaluate malaria control programs in which screening of malaria infection is necessary or as a confirmatory test for detecting malaria infection. If desired, the PET-PCR can be duplexed to detect genus and *P. falciparum* in one tube, albeit with some slight loss of sensitivity for the *P. falciparum-*specific primers. This is common with multiplex assays, which often require some compromise of the components and assay conditions to accommodate two or more primer sets which can lead to primer competition and loss of sensitivity. This caveat is not insurmountable as further improvements can be undertaken to address this issue. However, while duplexing these primer sets is a possibility, detection of mixed infections is not achievable because a positive result with the genus primer only indicates that Plasmodium spp. is present. Used together these two primer sets only indicates an infection with *P. falciparum* and does not necessarily indicate the lack of a mixed infection with other Plasmodium species. This is the same caveat encountered in many of the commonly used malaria RDTs that detect pan-Plasmodium antigens and the *P. falciparum* specific HRP-2 protein. Development of primers to detect non-*falciparum* species (*P. vivax, P. malariae, P. ovale* and *P. knowlesi*) will be required to make this method applicable in clinical laboratory settings.

Diagnostic tools for large scale field application need to be cheaper, robust, and easy to use. The PET-PCR assay described here meets these criteria and can be used for large scale screening in surveillance and epidemiological studies including as a confirmatory test in clinical laboratory settings.
